# Primary alveolar rhabdomyosarcoma of the brain: a case report

**DOI:** 10.1186/s13256-024-04471-w

**Published:** 2024-03-23

**Authors:** Layal Al Mahmasani, Marwan Najjar, Roula Hourany, Abeer Tabbarah, Sara Sinno, Nathalie Chamseddine, Reine Abou Zeidane, Ghid Amhaz, Bassem Youssef, Hazem I. Assi

**Affiliations:** 1 Division of Haematology-Oncology, Department of Internal Medicine, American University of Beirut Medical Center, Riad El Solh, P.O. Box: 11-0236, Beirut, 1107 2020 Lebanon; 2 Division of Neurosurgery, Department of Surgery, American University of Beirut Medical Centre, Beirut, Lebanon; 3 Department of Diagnostic Radiology, American University of Beirut Medical Centre, Beirut, Lebanon; 4 Department of Pathology, American University of Beirut Medical Centre, Beirut, Lebanon; 5 Department of Radiation Oncology, American University of Beirut Medical Centre, Beirut, Lebanon

**Keywords:** Primary alveolar rhabdomyosarcoma, Intracranial, Multidisciplinary approach

## Abstract

**Background:**

Primary brain rhabdomyosarcoma is a rare primary brain malignancy with few case reports. The vast majority of cases of primary brain rhabdomyosarcoma occur in pediatric patients, and immunohistochemistry can distinguish it from embryonal subtypes; however, few cases of primary brain rhabdomyosarcoma in adults have been reported in the literature.

**Case presentation:**

We report the case of a 26-year-old White male patient who was found to have primary brain alveolar rhabdomyosarcoma after developing headaches for several months. A brain MRI revealed a mixed cystic and solid tumor along the vermis of the cerebellum. The patient underwent a gross total surgical resection, which confirmed the diagnosis of alveolar rhabdomyosarcoma. Further staging workup for another primary focus or disseminated disease yielded negative results, confirming the diagnosis of primary alveolar rhabdomyosarcoma of the brain.

**Conclusion:**

The standard of care for managing this rare type of brain tumor involves surgery with adjuvant chemoradiotherapy. Further studies should be conducted for a better diagnostic and therapeutic understanding.

## Introduction

Primary brain rhabdomyosarcoma is a rare primary brain malignancy with scant case reports. While the majority of cases of primary brain rhabdomyosarcoma occur in pediatric patients, a few cases of primary brain rhabdomyosarcoma in adults have been reported in the literature. Myogenin immunostaining has been described as a useful marker of the alveolar subtype of rhabdomyosarcoma and as a tool for distinguishing it from the more common embryonal subtype [1]. We report a rare case of an adult who was diagnosed with primary brain alveolar rhabdomyosarcoma, for which an extensive immunohistochemistry workup and molecular profiling panel were done.

## Case report

**Fig. 1:**
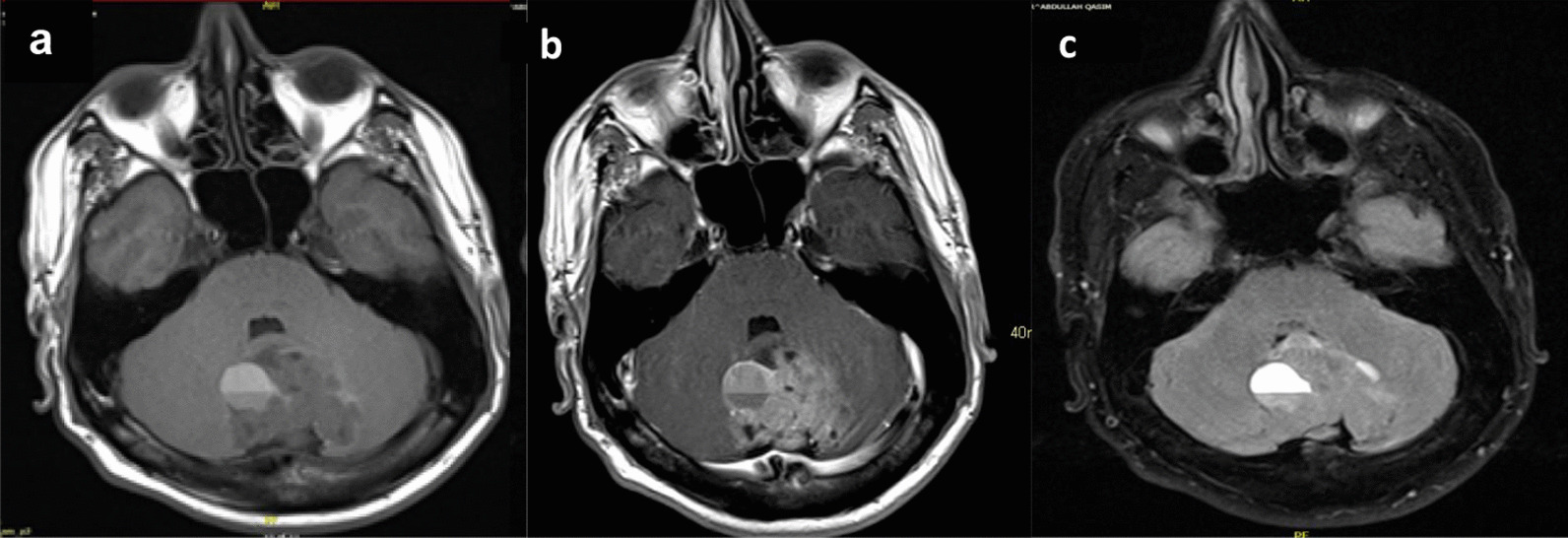
**a** axial non-enhanced T1, **b** axial enhanced T1 weighted image and c. axial FLAIR image of the brain. There is a predominantly T1 hypointense mass along the vermis, containing areas of T1 hyperintense fluid–fluid level. It shows strong heterogenous enhancement after contrast administration (**b**). There is no significant surrounding edema on FLAIR (**c**). It is compressing and displacing the fourth ventricle anteriorly

The patient was a 26-year-old White male with no known medical history who started to complain of headaches six months before presentation. The headache worsened over time and became more severe in the last month. No abnormal findings on physical examination were noted. No nausea, vomiting, unsteadiness, or any other associated signs or symptoms. An MRI of the brain (Fig. 1) showed a 4.5 × 4.3 × 3.9 cm mixed solid and cystic tumor along the surface of the vermis of the cerebellum. It contained multiple cystic areas with blood and a solid, strongly enhancing component. There was no surrounding vasogenic edema. It caused a mass effect on the fourth ventricle with secondary supratentorial hydrocephalus and CSF seepage. There was cerebellar tonsillar herniation and upward herniation of the cerebellum, with effacement of the quadrigeminal cistern. The radiological findings were suggestive of an ependymoma. An MRI of the spine was performed to complete the workup and revealed no abnormalities.

**Fig. 2:**
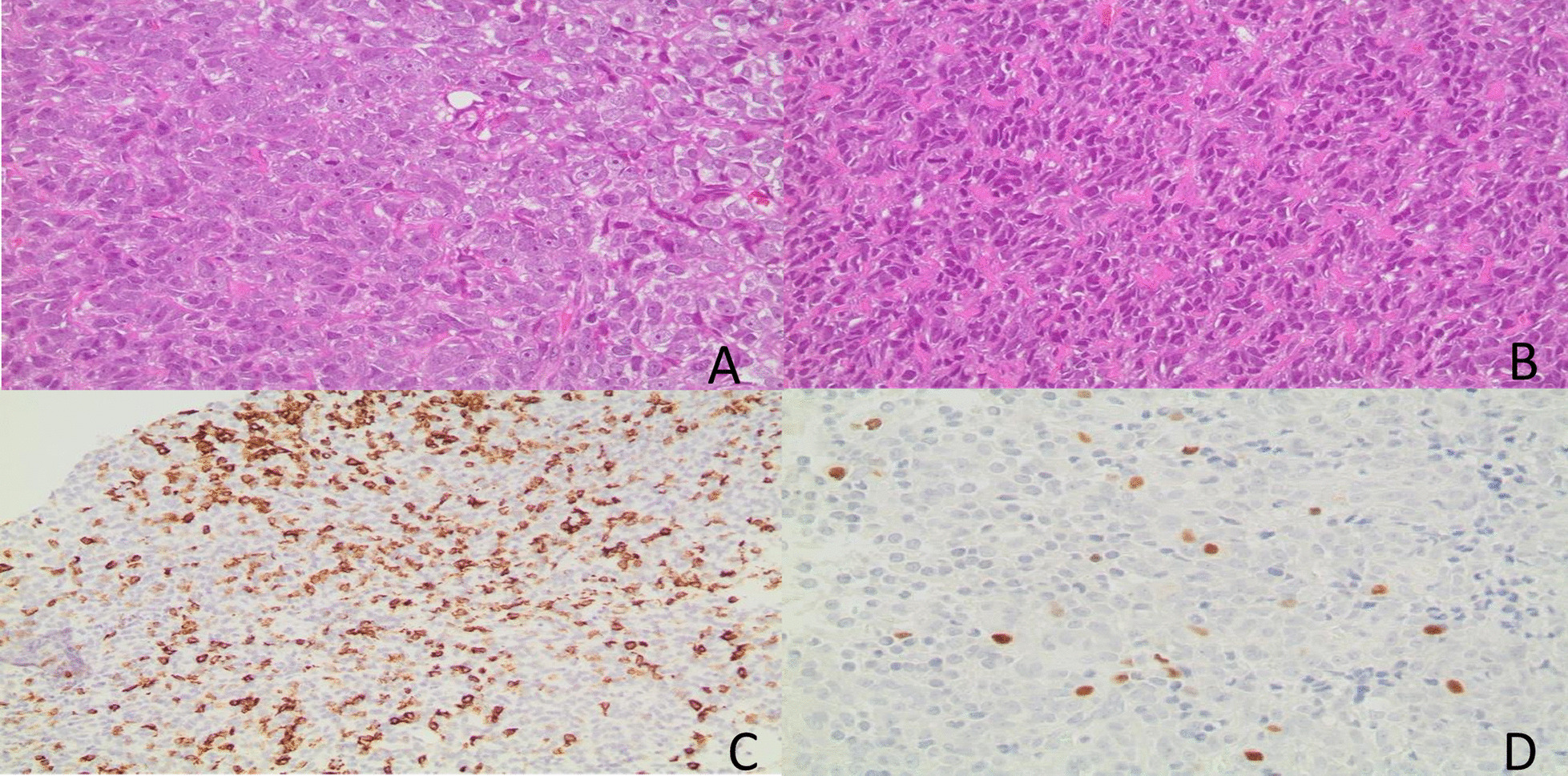
Alveolar rhabdomyosarcoma. **A** and **B** (H&E stain). The tumor is hypercellular and is composed of round cells with prominent nucleoli. Mitotic activity is brisk. **C** The tumor cells show strong positivity for Desmin. **D** Myogenin is positive in a subset of tumor cells

After case discussion and imaging review in a multidisciplinary meeting at our institution involving oncology, neurosurgery, pathology, radiation oncology, and radiology, the patient underwent surgery and gross total resection of the tumor after one week of presentation. Surprisingly, the pathology showed a high-grade neoplasm with immunophenotypic and molecular features of alveolar rhabdomyosarcoma. Histologic examination revealed a densely cellular neoplasm comprising round cells arranged in sheets. The tumor cells had round-to-oval nuclei and prominent nucleoli (Fig. 2A, B). The mitotic figures were elevated, and scattered foci of necrosis were identified. Entrapped islands of the neuropil were noted focally. Immunohistochemistry revealed that the tumor cells were negative for the glial markers GFAP and OLIG2. Desmin and myogenin were strongly expressed in a subset of tumor cells (Fig. 2C, D). The cells showed variable expression levels of BCOR, BCL2, BCL6, p53, INSM1, and synaptophysin. They were negative for BRAF V600E mutant protein, CAM5.2, EMA, H3K27M mutant protein, mutant IDH1, NeuN, S100, and SOX-10. H3K27 trimethylation was preserved. In addition, the nuclear expression of INI-1 and ATRX was retained. The Ki-67 proliferative index was elevated. A molecular oncogenic gene fusion panel detected a PAX3-INO80D fusion. Two PTEN mutations (p.V290fs and p.T319*) were identified using Next Generation sequencing (NGS). There were no mutations in IDH1, IDH2, BRAF, TP53, histone H3, NF1, NF2, DICER1, RB1, CDKN2A, ATRX, or TERT promoters. There were no reportable copy numbers. The overall findings were those of a high-grade neoplasm with immunophenotypic and molecular findings consistent with alveolar rhabdomyosarcoma. The staging workup, which included total-body CT scans and a testicular ultrasound, was negative for the primary disease. Therefore, we are dealing with a rare entity among adults called primary alveolar rhabdomyosarcoma of the brain. Our patient was scheduled to start radiation therapy in his home country, followed by systemic therapy (VDC-IE protocol). The patient was then lost to follow-up.

## Discussion

Rhabdomyosarcomas are common tumors of the head and neck region in children and rarely in adults. In addition, the intracranial localization of this tumor is rare. Among the few primary brain sarcomas, 70% arise in the pediatric population, and there have been limited cases reported in adults [2]. Primary intracranial sarcomas account for only 0.1–4.3% of all intracranial neoplasms and are more likely to be found in children [3]. Further classification of brain rhabdomyosarcoma can be based on the cell of origin, where intracranial rhabdomyosarcomas are more likely to arise from the brain substance and extend to the meninges, in contrast to meningeal rhabdomyosarcomas that arise from pluripotent mesenchymal cells of the meninges [4].

The majority of case reports in the literature describe cases of brain rhabdomyosarcoma of secondary origin, such as from the extremities, lungs, scalp, and genital tract. A few cases of primary intracranial alveolar rhabdomyosarcoma have been reported. A review of the literature identified 47 cases of primary cerebral rhabdomyosarcoma, of which 33 were children, and the majority were embedded in the surface of the brain and rarely entirely intraparenchymal or intraventricular [5]. Our patient was a rare case of primary cerebellar rhabdomyosarcoma in an adult patient.

The clinical and histological criteria currently used to classify rhabdomyosarcomas are complex, and their predictive power is limited. However, the role of molecular assays in the subclassification of patients with alveolar rhabdomyosarcoma by associating distinctive clinical patterns with the common and variant gene fusions has been shown to be helpful. For example, alveolar rhabdomyosarcoma is characterized by consistent chromosomal translocations and chimeric genes, PAX3-FKHR and PAX7-FKHR, which are expressed as novel fusion transcripts [6].

Following histological diagnosis and staging, there is still much debate regarding the optimal course of treatment. The inherent difficulty in discerning the cell of origin in rhabdomyosarcoma makes the selection of appropriate chemotherapy difficult and its usefulness debatable [7]. In addition, given the rarity of intracranial rhabdomyosarcoma and the heterogeneity of treatments, little could be concluded regarding the best therapeutic approach. However, the most common approach is to perform surgery first, followed by adjuvant radiation therapy and chemotherapy.

## Conclusion

Our patient is one of the few adults with primary brain rhabdomyosarcoma. Given the rarity of these tumors, a multidisciplinary approach is necessary. Currently, the safest surgical resection of the tumor, followed by adjuvant chemotherapy and radiotherapy, remains the logical approach to managing these tumors to maximize long-term survival. However, further studies are required to better understand the behavior of these tumors, identify an optimal therapeutic plan and standardize diagnostic immunohistochemistry and molecular profiling tests.

## Data Availability

The data presented in this study are available on request from the corresponding author. The data are not publicly available due to patient confidentiality pertaining to medical electronic health records from which our data were collected.
